# Colour As a Signal for Entraining the Mammalian Circadian Clock

**DOI:** 10.1371/journal.pbio.1002127

**Published:** 2015-04-17

**Authors:** Lauren Walmsley, Lydia Hanna, Josh Mouland, Franck Martial, Alexander West, Andrew R. Smedley, David A. Bechtold, Ann R. Webb, Robert J. Lucas, Timothy M. Brown

**Affiliations:** 1 Faculty of Life Sciences, University of Manchester, Manchester, United Kingdom; 2 School of Earth, Atmospheric and Environmental Sciences, University of Manchester, Manchester, United Kingdom; Charité - Universitätsmedizin Berlin, GERMANY

## Abstract

Twilight is characterised by changes in both quantity (“irradiance”) and quality (“colour”) of light. Animals use the variation in irradiance to adjust their internal circadian clocks, aligning their behaviour and physiology with the solar cycle. However, it is currently unknown whether changes in colour also contribute to this entrainment process. Using environmental measurements, we show here that mammalian blue–yellow colour discrimination provides a more reliable method of tracking twilight progression than simply measuring irradiance. We next use electrophysiological recordings to demonstrate that neurons in the mouse suprachiasmatic circadian clock display the cone-dependent spectral opponency required to make use of this information. Thus, our data show that some clock neurons are highly sensitive to changes in spectral composition occurring over twilight and that this input dictates their response to changes in irradiance. Finally, using mice housed under photoperiods with simulated dawn/dusk transitions, we confirm that spectral changes occurring during twilight are required for appropriate circadian alignment under natural conditions. Together, these data reveal a new sensory mechanism for telling time of day that would be available to any mammalian species capable of chromatic vision.

## Abstract 2

Environmental measurements and physiological recordings reveal that mice not only use changes in the intensity of sunlight to entrain their circadian clock, but also employ blue–yellow color discrimination to detect spectral changes associated with dawn and dusk.

## Author Summary

Animals use an internal brain clock to keep track of time and adjust their behaviour in anticipation of the coming day or night. To be useful, however, this clock must be synchronised to external time. Assessing external time is typically thought to rely on measuring large changes in ambient light intensity that occur over dawn/dusk. The colour of light also changes over these twilight transitions, but it is currently unknown whether such changes in colour are important for synchronising biological clocks to the solar cycle. Here we show that the mammalian blue–yellow colour discrimination axis provides a more reliable indication of twilight progression than a system solely measuring changes in light intensity. We go on to use electrical recordings from the brain clock to reveal the presence of many neurons that can track changes in blue–yellow colour occurring during natural twilight. Finally, using mice housed under lighting regimes with simulated dawn/dusk transitions, we show that changes in colour are required for appropriate biological timing with respect to the solar cycle. In sum, our data reveal a new sensory mechanism for estimating time of day that should be available to all mammals capable of chromatic vision, including humans.

## Introduction

The ability to predict and adapt to recurring events in the environment is fundamental to survival. Organisms across the living world achieve this using endogenous circadian clocks [1–3]. However, if such clocks are to fulfil their ethological function they need to be regularly reset to local time. This is achieved by sensory inputs that report changes in the physical environment providing a useful proxy for time of day. By far the best characterised of these input pathways is that recording the diurnal change in the overall quantity of light reaching the earth’s surface (irradiance). In the case of mammals, a dedicated retino-hypothalamic projection brings this visual information to the brain’s “master” clock in the suprachiasmatic nuclei (SCN) [4–7].

The retino-hypothalamic projection is formed by a unique class of retinal ganglion cells (RGCs), which are intrinsically photosensitive thanks to their expression of melanopsin [4]. Although these so-called ipRGCs can therefore entrain the clock even in the absence of the conventional rod and cone photoreceptors, all photoreceptor classes are capable of influencing the clock in intact animals [8–13]. This arrangement has previously been considered only insofar as it allows the clock to respond to changes in irradiance. However, the inclusion of cones in this pathway allows for the possibility that the clock could also receive information about changes in the spectral composition of light (colour) [14]. There has previously been speculation that such colour signals could provide a reliable method of telling time of day [3,15] but, to date, there has been no direct test of that possibility in mammals.

The ability to discriminate colour relies on comparing the relative activation of photopigments with divergent spectral sensitivities. In mammals, this task is achieved via differential processing of cone photoreceptor signals in the retina [16]. At least 90% of mammalian species are believed capable of this form of colour discrimination [17] which, with the exception of Old World primates, allows for dichromatic vision. Thus, most mammals express just two distinct classes of cone opsin, one maximally sensitive to short wavelengths (ultraviolet–blue) and a second with peak sensitivity to longer wavelengths (green–red) [18]. Here we show that, in mice, this primordial colour discrimination axis (equivalent to human blue–yellow colour vision) is an influential regulator of SCN activity, essential for appropriate circadian timing relative to the natural solar cycle.

## Results

### Changes in Colour across Twilight

We first set out to determine whether changes in spectral composition associated with the earth’s rotation could provide reliable information about solar angle that the circadian clock could use to estimate time of day. To this end, we obtained high resolution measurements of natural variations in spectral irradiance across multiple days (Manchester, August–October 2005, *n* = 36 d).

**Fig 1:**
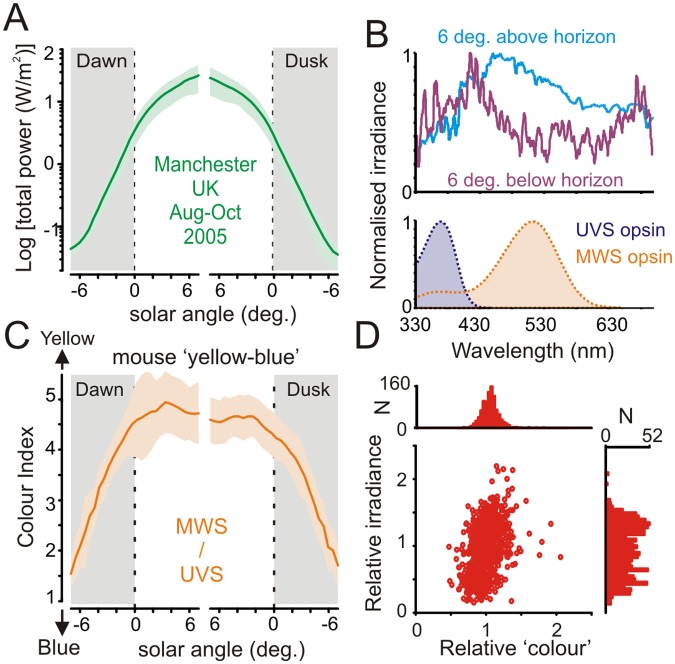
Spectral composition of ambient illumination is predictive of solar angle. (**A**) Mean (±SD) total optical power of ambient illumination around dawn/dusk as a function of solar angle relative to horizon (*n* = 36 d, Aug–Sep 2005; Lat.: 53.47, Long.: -2.23, Elevation 76 m). (**B**, top) Normalised mean spectral power distribution observed at solar angles ±6° relative to horizon. Note relative enrichment of short-wavelength light at negative solar angles. Bottom panel shows mouse ultraviolet and medium wavelength sensitive (UVS and MWS) cone opsin sensitivity profiles after correction for prereceptoral filtering. (**C**) Mean (±SD) “yellow–blue” colour index (effective activation of MWS/UVS opsin) as a function of solar angle around dawn/dusk. (**D**) Relationship between colour and irradiance (see Methods for definition), corrected according to mean for each solar angle (*n* = 994; -7 to 0° in 0.5° bins × 71 dawn/dusk observations; for clarity, six observations with especially high relative brightness (2.6–4.5) but normal colour (1.3–1.5) are not shown in the scatter plot). Note tighter distribution for relative “colour” versus irradiance. The data used to make this figure can be found in S1 Data.

As expected, these measurements revealed highly predictable changes in both irradiance and spectral composition as a function of solar angle (Fig 1A and 1B). In particular, we observed a progressive enrichment of short-wavelength light across negative solar angles: a result of the increasing amount of ozone absorption and consequent Chappuis band filtering of green–yellow light when the sun was below the horizon [14].

We next calculated the extent to which this change in spectral composition was detectable to the mammalian visual system. Taking the mouse as a representative species, we employed previously validated approaches to quantify the relative excitation of its ultraviolet and medium wavelength sensitive (UVS/MWS) cone opsins [12,19,20]. This analysis revealed a robust change in the ratio of excitation between the two pigments (Fig 1C) that would constitute substantial changes in apparent colour along the blue–yellow axis. These changes were restricted to the twilight transition, with the UVS:MWS ratio fairly invariant throughout the day, indicating that measuring the change in colour could provide a useful method of tracking the progression of dawn and dusk.

To ascertain how reliably this blue–yellow colour signal alone could be used to estimate phase of twilight, relative to simple measures of irradiance, we next compared the day-to-day variability of colour and irradiance measurements across our dataset (Fig 1D). Surprisingly, we found that colour was in fact more predictive of sun position across twilight (-7 to 0° below horizon) than was irradiance (78.5 ± 0.1% versus 75.8 ± 0.1% of variance explained by solar angle; mean ± SD). Accordingly, for any fixed solar angle, the range of observed colour values was considerably more tightly clustered than those for irradiance. These observations most likely reflect the fact that cloud cover can change overall brightness quite dramatically, but exerts only relatively minor effects on spectral composition.

### Colour Coding in the SCN

Importantly, then, measuring colour could provide a more reliable estimate of the approach of night or day than measuring irradiance. However, while the mammalian circadian clock is certainly known to respond to diurnal variations in irradiance [8–13], there has been no investigation of whether the SCN also receives colour signals. Accordingly, we next asked whether the central clock showed electrophysiological responses to changes in colour by recording extracellular activity in the mouse SCN.

In order to identify colour-sensitive cells, we set out to generate test stimuli which differentially modulated the UVS and MWS mouse cone opsins. While, in principle, producing such stimuli is straightforward, the close spectral sensitivity of mouse opsins makes it difficult to achieve this aim without concomitant changes in the activation of rods and/or melanopsin. To circumvent this problem, we employed a well-validated transgenic model in which the native mouse MWS opsin is replaced by the human long-wavelength sensitive (LWS) opsin (*Opn1mw* ^*R*^; [8,12,19]). Cones in these animals develop and function normally, with LWS opsin expression entirely recapitulating that of the native MWS opsin [21]. Importantly, however, the resultant shift in cone spectral sensitivity in *Opn1mw* ^*R*^ mice facilitates the generation of stimuli that provide selective modulation of individual opsin classes [22].

Using this *Opn1mw* ^*R*^ model, we first established a background lighting condition (using a three-primary LED system), whose spectral composition recreated a wild-type mouse’s experience of natural daylight (S1A Fig). We next designed a set of manipulations of this background spectrum that allowed us to modulate excitation of one or both cone opsins without any concomitant change in rod or melanopsin activation. Under these conditions, we were then able to unambiguously distinguish colour-sensitive neurons based on the following criteria: (1) the presence of larger responses to chromatic versus achromatic changes in cone excitation and (2) responses of opposite sign to selective activation of UVS and LWS opsin in isolation (i.e., excitatory/ON versus inhibitory/OFF).

**Fig 2:**
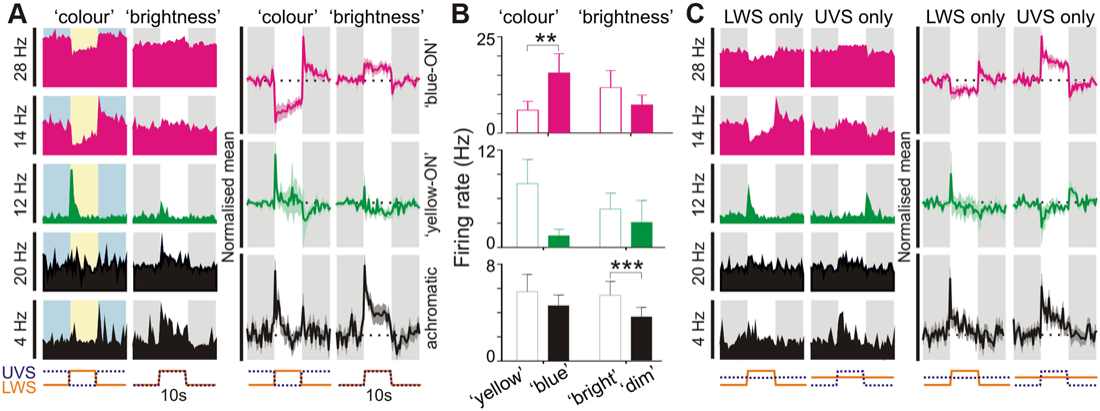
Colour opponent responses in suprachiasmatic neurons. (**A**: left) Example responses of 5 SCN neurons to stimuli modulating UVS and LWS opsin excitation in antiphase (“colour”) or in unison (“brightness”; both 70% Michelson contrast). Individual cells were preferentially excited by blue–yellow transitions (“blue ON”; pink traces), yellow-blue transitions (“yellow ON”; green traces) or dim-bright transitions (“achromatic”; black traces). Shaded areas represent blue/yellow or dim/bright stimulus phase; *y*-axis scale bars reflects peak firing rate in spikes/s; *x*-axis scale bars indicate temporal profile of UVS/LWS opsin excitation. (**A**: right) Normalised mean (±SEM) change in firing for cells classified as “blue-ON”, “yellow-ON” or achromatic (*n* = 13, 4 and 26 respectively). Conventions as above. (**B**) Mean (±SEM) firing rates of SCN cell populations tested with colour and brightness stimuli immediately following transitions (0–500 ms) from “blue”–”yellow”/”dim”–”bright” or vice versa. Data were analysed by paired *t* test; *** *p*<0.001, ** *p*<0.01. (**C**: left) Responses of cells from **A** to selective modulation of LWS or UVS opsin excitation, indicating “blue”-ON/”yellow-OFF”, “yellow-ON”/”blue-OFF” or non-opponent responses (conventions as in **A**). (**C**: right) Normalised mean (±SEM) change in firing for SCN cell populations evoked by LWS and UVS opsin isolating stimuli. Note, normalisation and scaling for data in **A** and **C** is identical. The data used to make this figure can be found in S2 Data.

To achieve the largest possible change in colour, we started by selectively modulating UVS and LWS cone opsin excitation in antiphase (“colour”; S1B Fig). We then compared responses to this stimulus with those elicited by one in which the change in UVS and LWS opsin activation occurred in unison (“brightness”; S1B Fig). Any spectrally opponent cells should be more responsive to the “colour” as opposed to “brightness” condition. We found that 17/43 SCN units (from 15 mice) that responded to these stimuli showed a significant preference for the pair in which UVS and LWS activation was modulated in antiphase (Fig 2A and 2B; paired *t* test, *p*<0.05, *n* = 17). As there were an additional 26 visually responsive units that did not respond to either of these analytical stimuli (paired *t* tests, *p*>0.05), these data indicate that at least one quarter of light-responsive SCN neurones show chromatic opponency.

Interestingly, we found that cone inputs exerted a much more powerful influence over the firing activity of cells exhibiting a preference for chromatic stimuli relative to than achromatic cells (Fig 2B; absolute change for responses of chromatic cells = 8.1 ± 2.3 spikes/s versus 1.9 ± 0.5 spikes/s for achromatic cells, *n* = 17 and 26 respectively; *t* test: *p*<0.01). Moreover, we found that the spiking activity for the majority (13/17) of colour-sensitive SCN neurons was highest during the stimulus phase biased towards UVS opsin activation (Fig 2A), and that these cells exhibited especially robust and sustained changes in firing (Fig 2A and 2B).

Our data above therefore indicate that cone inputs constitute a dominant influence on the firing activity of colour-sensitive SCN neurons and that most of these cells exhibit blue-ON/yellow-OFF colour opponency. We confirmed this by selectively modulating brightness for each of these cone opsins independently (stimulus shown in S2A Fig); as expected, these cells reliably increased firing in response to selective increases in UVS opsin activation and decreased firing following increases in LWS opsin activation (Fig 2C). Conversely, the remaining colour-sensitive cells exhibited the opposite preference (yellow-ON/blue-OFF; Fig 2C).

An aspect of mouse retinal organisation that poses a challenge to colour vision is that most cones in this species co-express UVS and MWS opsin [23]. The exceptions are rare “primordial S-cones” that only express UVS opsin [24] and peripheral cones that may express either pigment alone [25]. One might expect that chromatic opponency would rely on comparisons between these rare single pigment cones. If this were the case, then responses to LWS- and UVS-specific stimuli of defined contrast should be insensitive to changes in the spectral composition of the background light. In fact, we found that this was not the case (S2A–S2C Fig), indicating involvement of the more common opsin co-expressing cones in the chromatic responses of SCN neurons.

By contrast with chromatic SCN neurons, none of the cells identified as achromatic exhibited any overt OFF response to selective activation of either UVS or LWS cone opsin. Instead, these achromatic cells exhibited pure ON responses to stimuli targeting one or both opsin classes, such that on average the population showed little bias towards UVS/LWS opsin-driven responses under background spectra resembling natural daylight (Fig 2C). Adjusting the background spectra to equalise basal activation of the two cone opsins skewed responses in favour of UVS opsin, however (S2D Fig), consistent with previous suggestions that ipRGCs are relatively enriched in the UVS opsin-biased dorsal retina [26].

**Fig 3:**
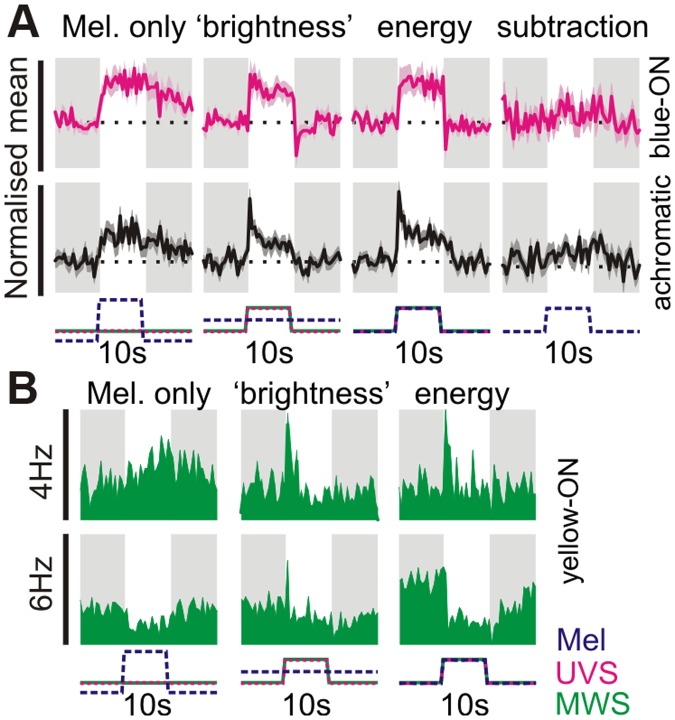
Melanopsin signals influence both colour- and brightness-sensitive cells. (**A**) Normalised mean (±SEM) response of blue-ON colour-sensitive (*n* = 13) and achromatic cells (*n* = 23 tested) to stimuli targeting melanopsin and/or cones. Melanopsin-isolating stimuli presented a 92% Michelson contrast change (~1.4 log units), all other stimuli were 70% Michelson contrast (see S1A Fig for details of “colour” and “brightness” stimuli). The energy condition reflects a spectrally neutral modulation in light intensity, providing 70% Michelson contrast for all retinal opsins. Far right panels reflect the predicted melanopsin contribution to the 70% energy condition (obtained by subtracting the responses to UVS + LWS only −”brightness”). Responses were normalised on a within-cell basis across all three stimulus conditions and are plotted on the same scale to highlight relative response amplitude. *X*-axis scale bars indicate temporal profile of UVS/LWS opsin and melanopsin excitation. (**B**) Example responses of yellow-ON colour-sensitive cells (bottom panels) to stimuli targeting melanopsin and/or cones or melanopsin. Melanopsin-isolating contrast had more heterogeneous effects in yellow-ON cells, with 1/4 cells exhibiting a reduction in firing and 2 cells displaying no obvious response (not shown). Conventions as above except that data are presented as raw firing rates. *Y*-axis scale bars represent peak firing in spikes/s. The data used to make this figure can be found in S3 Data.

We next asked whether colour opponent cells also received irradiance information from the melanopsin-expressing ipRGCs that dominate retinal input to the SCN [4,6,7]. To this end, we used changes in spectral composition to selectively modulate melanopsin excitation (see Methods; 14/15 mice above tested with these stimuli). When presented with large steps in melanopsin excitation (92% Michelson contrast) generated in this way, “blue”-ON cells showed slow and sustained increases in firing (Fig 3A; peak response = 3.2 ± 0.8 spikes/s above baseline; paired *t* test, *p*<0.01, *n* = 13), as previously described for melanopsin-driven responses [8,27,28]. The behaviour of the rare “yellow” ON cells to this stimulus was variable (Fig 3B; *n* = 4), while colour-insensitive cells showed the expected excitatory response (Fig 3A; peak response = 1.8 ± 0.2 spikes/s above baseline; paired *t* test, *p*<0.01, *n* = 23). These data therefore reveal that both chromatic and achromatic cells have access to melanopsin-dependent information about irradiance.

Of note, for the smaller changes in opsin excitation applied above (70% Michelson; ~0.75 log units), we found that the inclusion of melanopsin contrast had little impact on the integrated cellular response. Thus for both chromatic and achromatic populations, responses evoked by spectrally neutral increases in irradiance (“energy”) were very similar in magnitude to those observed where changes in irradiance were restricted to just cone opsins (Fig 3A; subtraction: energy − “brightness”). This was true even for steady-state components of the SCN response (last 1 s of step)—we found no significant difference in responses to two conditions (paired *t* test; *p*>0.05 for both blue-ON and achromatic populations). Thus SCN responses to relatively modest changes in light intensity and/or spectral composition are, in fact, dominated by those originating with cones.

**Fig 4:**
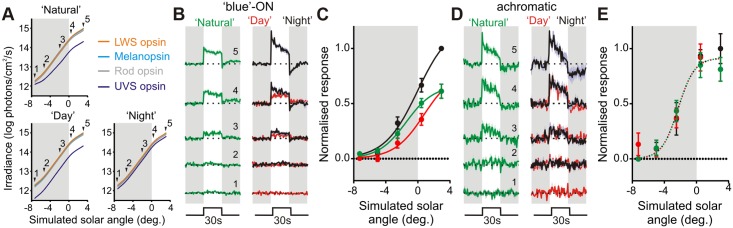
Colour-signals control irradiance coding in suprachiasmatic neurons. (**A**) Stimuli used to examine twilight coding: top panel indicates natural change in effective photon flux for each mouse opsin as a function of solar angle (0° represents sunrise/sunset), indicated points were recreated using a three-primary LED system. Note: values for LWS opsin stimulation were chosen to replicate those calculated for the wild-type MWS opsin under natural conditions. Bottom panels indicate control stimuli, which replicated the “natural” change in irradiance but lacked changes in colour (UVS opsin excitation held at a constant ratio relative to LWS, to mimic “day” or “night” spectra). (**B**) Mean (±SEM) normalised responses of blue-ON cells (*n* = 9) to 30-s light steps recreating the indicated stages of twilight. Responses were normalised on a within-cell basis according to the largest response observed across all three stimulus sets. (**C**) Initial (0–10 s) responses of cells from **B** as a function of simulated solar angle, fit with four-parameter sigmoid curves. Note influence of twilight spectral composition on the solar angle response curve (F-test for difference in curve parameters; *p* = 0.009; direct comparisons between each pair of curves also revealed significant differences *p*<0.05). (**D and E**) Responses of achromatic cells (*n* = 8), conventions as in **B** and **C**. Achromatic cell responses to the three stimulus sets were statistically indistinguishable (F-test; *p* = 0.72). The data used to make this figure can be found in S4 Data.

How then do chromatic and irradiance responses interact to encode time of day under more natural conditions? To address this question, we produced stimuli that recreated, for *Opn1mw* ^*R*^ mice, the change in irradiance and colour experienced by wild-type (green cone) mice across the twilight to daylight transition (Fig 4A). We presented these as discrete light steps from darkness, to simulate the challenge in telling time of day faced by a rodent emerging from a subterranean burrow to sample the light environment. Due to their scarcity, we were unable to determine the behaviour of yellow-ON cells under these conditions. However, blue ON cells reliably exhibited a near linear increase in firing rate as a function of simulated solar angle (Fig 4B and 4C; *n* = 9 from 7 mice), indicating that their sensitivity is well suited to track changes in colour/irradiance occurring across the twilight to daylight transition. Interestingly, the range of solar angles to which these neurons responded was substantially greater than that for achromatic cells recorded in the same set of mice (Fig 4D and 4E; see also S3 Fig; *n* = 8) indicating that they may be an especially important source of temporal information for the clock around twilight.

To determine the extent to which this ability of blue-ON cells to encode solar angle relied upon their chromatic opponency, we next presented stimuli that recreated the natural change in irradiance over twilight but in which colour was invariant. Two versions of these stimuli were produced, in which colour was fixed either to that at the lowest solar angle for which data was available (“night”) or to that recorded in daylight (“day”; Fig 4A). Whereas achromatic cells were unable to distinguish between these two stimulus sets (Fig 4D and 4E; F-test, *p* = 0.72), the relationship between solar angle and blue-ON cell firing rate was consistently disrupted under these conditions (Fig 4B and 4C; F-test, *p* = 0.009; see also S3A Fig). Thus, firing was reliably higher for “night” and lower for “day” conditions than appropriate for that time of day. These effects are consistent with the blue-ON nature of the chromatic units and confirm that these cells employ a combination of colour and irradiance signals in order to encode time of day.

### Colour Sets Circadian Phase

These electrophysiological recordings indicate a significant fraction of neurons in the SCN convey information about changes in spectral composition occurring during natural twilight. We hypothesised, therefore, that by improving the SCN’s ability to estimate solar angle, activation of the colour mechanism would influence the phasing of circadian rhythms under natural conditions. To determine whether this was indeed the case, we scaled up our twilight stimuli to produce an artificial sky that could be presented to freely moving mice over many days in their home cage. We aimed then to compare the phase of circadian rhythms (assayed using body temperature telemetry) under exposure to lighting conditions that recreated natural changes in irradiance across dawn/dusk transitions, with or without the associated alterations in colour (S4 Fig; “irradiance only” twilight replicated “night” spectral composition). To maximise our ability to detect changes in phasing under these conditions, we modelled the temporal profile of these photoperiods on the extended twilight of a northern-latitude summer (S4C Fig). To allow us to readily separate irradiance and colour elements, we undertook these experiments in *Opn1mw* ^*R*^ mice. Importantly, however, we designed the stimuli to recreate the change in colour across twilight that is experienced by normal, wild-type mice.

**Fig 5:**
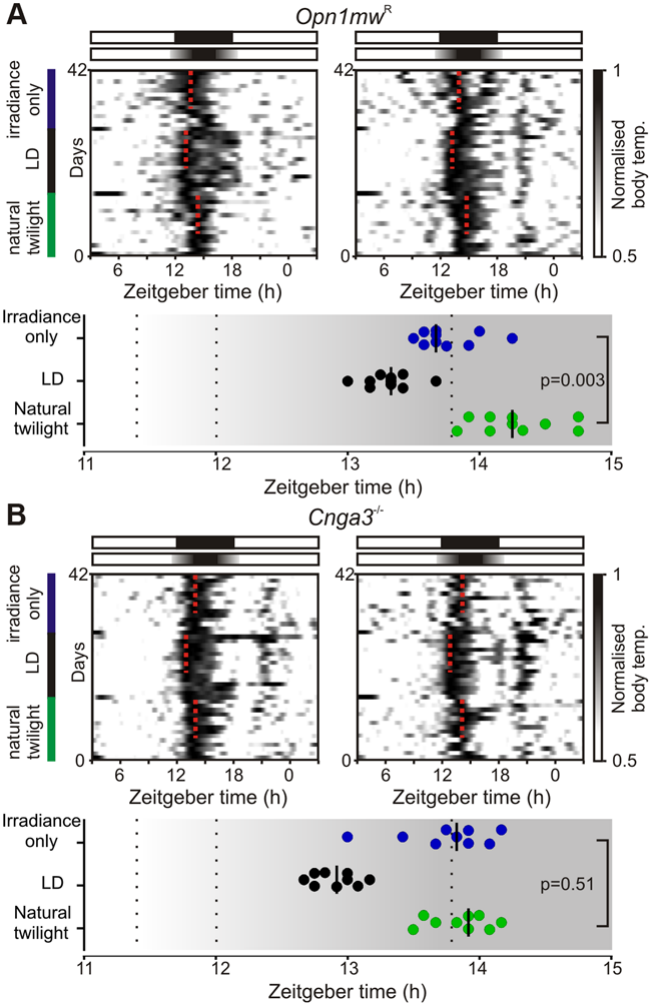
Colour changes associated with natural twilight influence circadian entrainment. **(A)** Top: Example body temperature traces from two *Opn1mw* ^R^ mice. Mice were exposed to sequential 14 d epochs of (i) simulated “natural” twilight (replicating natural changes in irradiance and colour during a northern-latitude summer), (ii) 18:6 square wave LD cycle, and (iii) a twilight photoperiod which lacked changes in colour (irradiance profile identical to “natural” but relative cone opsin excitation fixed to mimic night spectra). Dotted red lines indicate timing of peak body temperature from last 9 d in each photoperiod. Bottom plot indicates timing of peak body temperature for each individual (*n* = 10); bars represent median. Temperature cycles were significantly phase-advanced under the irradiance-only versus natural twilight (paired *t* test; *p* = 0.003). (**B**) Mice lacking functional cone phototransduction (*Cnga3* ^-/-^) exhibit identical phase of entrainment under both photoperiods (conventions as in **A**; paired *t* test; *p* = 0.51, *n* = 9) with peak body temperature occurring significantly earlier versus wild-type mice under natural but not irradiance-only twilight (unpaired *t* tests, *p* = 0.005 and 0.91 respectively). The data used to make this figure can be found in S5 Data.

We found that the inclusion of colour significantly altered the phase of circadian entrainment. Peak body temperature occurred consistently later when irradiance and colour elements of twilight were included compared to the irradiance signal alone (Fig 5A; 31 ± 8 min; paired *t* test, *p* = 0.003; *n* = 10). This distinction was absent in mice lacking cone phototransduction (*Cnga3* ^-/-^, [29,30]; Fig 5B; 6 ± 9 min; paired *t* test, *p* = 0.51; *n* = 9), confirming that it originated with cone-dependent colour coding, rather than any differences in the pattern of rod/melanopsin activation between the two photoperiods.

**Fig 6:**
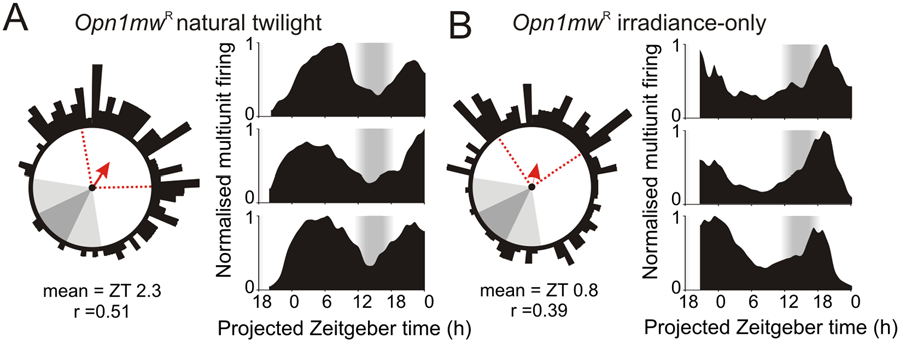
Twilight spectral composition regulates photoperiodic encoding in the suprachiasmatic nuclei. (**A**–**B**) Phasing of SCN firing rhythms from ex vivo multielectrode array recordings of *Opn1mw* ^R^ mice housed under natural (**A**) or irradiance-only (**B**) twilight photoperiods. Left panels show Rayleigh vector plots for peak firing activity (*n* = 124 and 170 SCN electrodes from seven and six slices in **A** and **B** respectively). Grey-shaded areas correspond to timing of night/twilight transitions, red dotted lines indicate central 50% of the data distribution, arrows indicate mean vector direction. Right panels show representative multiunit traces. Consistent with body temperature data (Fig 5), SCN activity peaks later in mice housed under “natural” relative to irradiance-only twilight (*p*<0.001 based on bootstrap percentiles). The data used to make this figure can be found in S6 Data.

As further confirmation that these differences in body temperature cycles reflected an action on the timing of central clock output, we also monitored SCN firing rate rhythms in a subset of mice via ex vivo multielectrode array recordings. We and others have previously shown that the distribution of daily electrical activity patterns among individual SCN neurons encodes photoperiod duration, resulting in broad phase distributions under summer days [31,32]. Consistent with this work, peak multiunit firing (sampled across small groups of neurons) in the ex vivo SCN of twilight-housed mice was widely distributed across recording epochs corresponding to projected day. Importantly, in line with our body temperature data, this distribution was centred around the middle of the projected day for *Opn1mw* ^*R*^ mice exposed to “natural” twilight (Fig 6A; *n* = 124 SCN electrodes from seven slices) but shifted substantially earlier when mice were housed under twilight that lacked changes in colour (Fig 6B; *p*<0.001, bootstrap percentiles; *n* = 170 SCN electrodes from six slices). A similarly early phase of peak SCN electrical activity was also observed in slices prepared from *Cnga3* ^-/-^ individuals housed under natural twilight (S5 Fig; *p*<0.001 versus *Opn1mw* ^*R*^, bootstrap percentiles), confirming that the cone-dependent colour signal is indeed required for appropriate biological alignment with twilight. We also found that, across the three groups, *Opn1mw* ^*R*^ mice exposed to “irradiance-only” twilight exhibited a significantly broader distribution of SCN phasing (Brown-Forsythe test, *p* = 0.01), suggesting that the inappropriate cone signals under this photoperiod partially impair SCN synchrony.

## Discussion

Here we demonstrate that the mammalian clock has access to information about not just the amount but also the spectral composition of ambient illumination, in the form of a cone-dependent colour opponent input that reports blue–yellow colour. The idea that chromatic signals associated with twilight might provide important cues for circadian photoentrainment has been proposed previously [3,15]. However, the significant technical challenges inherent in distinguishing the influence of changes in colour versus brightness have left the specific role of colour untested, until now.

Our work thus represents the first demonstration that colour-opponent signals influence the circadian clock in any mammalian species. It is clear, from the long history of housing animals under artificial lighting, that colour signals are not necessary for circadian entrainment per se. However, our data indicates that most mammals could use colour [18,33–35] to provide additional information about sun position, above that available from simply measuring irradiance. Our entrainment experiments likely underestimate the importance of that colour signal under field conditions as they lack the daily variation in cloud cover that makes irradiance-alone a less reliable indicator of time of day. Nevertheless, even under these conditions, we find a significant impact of the twilight spectral change on the phasing of entrained rhythms. This reveals that spectral opponency contributes to the most fundamental function of the entrainment mechanism, ensuring correct timing of physiological and behavioural rhythms.

Given the nature of the change in spectral composition, we might expect that it would be available to any species capable of comparing the activity of short with middle/longer wavelengths. Our own subjective experience is that the event is detectable to humans, and a chromatic opponency equivalent to that described here could account for previous reports of subadditivity for polychromatic illumination in human melatonin suppression [36,37]. It is also noteworthy that the majority of mammalian species have retained the short and mid-/long-wavelength cone opsins required to detect changes in spectral composition associated with twilight (for a detailed discussion of the exceptions to this rule see [17,18]). Similarly, earlier studies have identified the capacity for blue–yellow colour discrimination in the pineal/parietal organs of a number of non-mammalian vertebrates, including reptiles, amphibians, and fish (for review see [38]). By directly influencing melatonin secretion, chromatic signals are thus presumably also a key component of the neural mechanisms responsible for appropriate alignment of non-mammalian physiology relative to dawn and dusk. Alongside our present data, it appears then that the use of colour as an indicator of time of day is an evolutionarily conserved strategy, perhaps even representing the original purpose of colour vision.

The specific sensory properties of the circadian photoentrainment mechanism in mammals have long remained a subject of debate [2]. SCN neurons are known to receive input from all major classes of retinal photoreceptor [8–13]. However, since “cone-only” mice do not reliably entrain to conventional light–dark cycles, current models posit that photoentrainment is primary driven by a combination of rod and melanopsin inputs [11,12]. By contrast, the proposed role of cones has been to allow the clock to track relatively high frequency changes in light—a signal that does not appear to play much role in circadian entrainment under conditions most commonly employed in the laboratory (but see [12]). Our data thus establish an important new role for cones in photoentrainment, one which would not be apparent under standard laboratory conditions but will act as an essential regulator of biological timing in more natural settings.

Insofar as most retinal input to the clock is provided by ipRGCs [4,6,7] the appearance of colour opponency in this subset of retinal ganglion cells would provide a simple explanation for the chromatic responses of SCN neurons observed here. Colour opponency has not yet been documented in mouse ipRGCs [39], but has been reported in primates [40] (although it is unknown whether any of these cells project to the SCN). Interestingly, the dominant form of spectral opponency we observe here in the mouse SCN (blue-ON/yellow-OFF) is opposite to that reported for primate ipRGCs and, most recently, for chromatic response of the pupil in humans [41]. While this would, by no means, rule out a role for chromatic influences on the human circadian system, it is also currently unclear whether such yellow-ON/blue-OFF responses are a characteristic feature of all primate ipRGCs. Indeed, such behaviour certainly appears inconsistent with the sensory properties of human melatonin regulation, which seems to exhibit a short- rather than long-wavelength bias [42].

Of course, alternative possibilities to that outlined above are that colour information reaches SCN neurons via the small number of non-melanopsin-expressing RGC inputs or is generated by a mechanism distinct from the conventional retinal colour processing circuitry. Previous work indicates that asymmetries in the gradient of cone opsin expression in the mouse retina could impose an indirect form of chromatic bias for stimuli larger than the cells’ receptive field [43]. Alternatively, opponent responses in the SCN may be generated centrally, e.g., via local processing or indirect visual input from the intergeniculate leaflet. Indeed, based on our identification of a rare yellow-ON cell exhibiting inhibitory responses to melanopsin contrast, we speculate that central processing could contribute to at least some of the responses reported here.

Regardless of their biological origin, chromatic signals provide the SCN with additional information about solar angle, above that available from measuring brightness alone, allowing the clock to appropriately time its output under natural photoperiods. Based on the widespread capacity for colour vision among mammals (and the previous identification of colour opponent ipRGCs in primates), we suggest related mechanisms are likely to be broadly applicable across many mammalian species.

## Methods

### Animals

All animal use was in accordance with the Animals (Scientific Procedures) Act of 1986 (United Kingdom). Electrophysiological experiments were performed under urethane anaesthesia; other procedures were conducted under isfluorane anaesthesia. Unless otherwise stated, animals used in this study (homozygous *Opn1mw* ^R^ and *Cnga3* ^-/-^ mice) were housed under a 12-h dark/light cycle at a temperature of 22°C with food and water available ad libitum.

### Environmental Measurements

Spectral irradiance measurements (280–700 nm, 0.5 nm bins) were collected in Manchester, UK (Lat.: 53.47, Long.: -2.23, Elevation 76 m) every minute across the solar cycle using a METCON diode array spectroradiometer contained within a temperature stabilised weatherproof housing. The global entrance optics was levelled and mounted at a rooftop monitoring site, providing a horizon relatively clear of obstructions, the entrance optics being connected to the spectrometer by way of a 600 μm diameter 5 m long optical fibre. Instrument calibrations were carried out with reference to spectral irradiance standards, traceable to NIST (National Institute of Standards and Technology, United States). Instrument dark counts were observed to be spectrally flat and were removed by subtracting the mean value for wavelengths <290 nm (where no ground-level solar signal is present).

### Environmental Data Modelling

Data analysed were spectral irradiance measurements collected between 31 August and 14 October 2005 (41 d). Due to gaps in the data collection record, we were able to extract from these 71 complete dawn/dusk transitions (from 36 d). No attempt was made to select data on the basis of weather condition although the period was broadly representative in comparison to relevant climatological averages.

For each twilight transition, we first calculated the average spectral irradiance profile as a function of solar angle relative to the Horizon (0.5° bins, 2–5 measurements/bin). We restricted this analysis to solar angles greater than 7° below the horizon, since our detector was specifically optimised to obtain measurements across light intensities encountered through civil twilight to daytime (making night-time measurements less reliable). We next converted these spectral irradiance profiles into effective photon fluxes as experienced by mouse opsin proteins, using established and validated procedures [19,20,22] based on Govardovski visual pigment templates [44] and published values for mouse lens transmission [45]. Calculations presented in the manuscript were based on the following peak sensitivity (λmax): UVS cone opsin-365 nm, Melanopsin-480 nm, Rhodopsin-498 nm, MWS cone opsin 511 nm.

The resulting series” of photon flux versus solar angle values for each opsin were then analysed individually or in combination (additive or as ratios). Specifically, we calculated the percentage of variance for the dataset in question that was explained by sun position, using the following calculation (with *N* representing the total number of data points, *K* the number of dawn/dusk observations and *P* the number of solar angle bins):

$$\text{Var}|\theta =100K\frac{\sum_{h=1}^{P}{\left({\bar{X}}_{h}-\bar{X}\right)}^{2}}{\sum_{i=1}^{N}{\left(X_{i}-\bar{X}\right)}^{2}}$$

Since there was no apparent difference in photon flux versus solar angle profiles obtained during dawn or dusk transitions, we pooled these data for the above analysis, treating each as an independent observation.

For comparisons of colour versus irradiance based estimates of solar angle (Fig 1D and associated text), irradiance was defined as effective photon flux at UVS+MWS cone opsins. Values obtained using other mouse opsins (singly or in combination) produced essentially identical results. For the aforementioned comparisons, estimates of mean and standard deviation for Var|θ were obtained based on bootstrap replicates (every possible combination of 69 out of the total 71 dawn/dusk observations). Similar analysis to those described above, but performed using only observations taken at either dawn or dusk also produced essentially identical results. Calculations of ““blue–yellow”“colour index (Fig 1C and 1D) were based on the ratio of MWS:SWS cone opsin activation ([MWS+LWS]/SWS for human visual system).

### In Vivo Electrophysiological Recordings

Urethane (1.55 g/kg) anaesthetised adult (60–120 d) male *Opn1mw* ^*R*^ mice were prepared for stereotaxic surgery as previously described [8]. Recording probes (Buszaki 32L; Neuronexus, MI, US) consisting of four shanks (spaced 200 μm), each with eight closely spaced recordings sites in diamond formation (intersite distance 20–34 μm) were coated with fluorescent dye (CM-DiI; Invitrogen, Paisley, UK) and then inserted into the brain 1 mm lateral and 0.4 mm caudal to bregma at an angle of 9° relative to the dorsal-ventral axis. Electrodes were then lowered to the level of the SCN using a fluid-filled micromanipulator (MO-10, Narishige International Ltd., London, UK).

After allowing 30 min for neural activity to stabilise following probe insertion, wideband neural signals were acquired using a Recorder64 system (Plexon, TX, US), amplified (x3000) and digitized at 40 kHz. Action potentials were discriminated from these signals offline as “virtual”-tetrode waveforms using custom MATLAB (The Mathworks Inc., MA, US) scripts and sorted manually using commercial principle components based software (Offline sorter, Plexon, TX, US) as described previously [46].

Surgical procedures were completed 1–2 h before the end of the home cage light phase, such that electrophysiological recordings spanned the late projected day-early projected night, an epoch when the SCN light response is most sensitive. Cells were initially characterised as light responsive on the basis of responses to bright mono and polychromatic light steps (10–30 s dur.; intensity >10^14^ photons/cm^2^/s). Once visual responsiveness was confirmed, experimental stimuli were applied as described below. Following the experiment, accurate electrode placement was confirmed histologically as described previously [8]. Projected anatomical locations of light response units reported in this study are presented in S6 Fig.

### Visual Stimuli

All visual stimuli were delivered in a darkened chamber from a custom built source (Cairn Research Ltd, Kent, UK) consisting of independently controlled UV, blue and amber LEDs (λmax: 365, 460, and 600 nm respectively). Light was combined by a series of dichroic mirrors and focused onto a 5 mm diameter piece of opal diffusing glass (Edmund Optics Inc., York, UK) positioned <1 mm from the eye (contralateral to the recording probe for SCN recordings). LED intensity was controlled by a PC running LabView 8.6 (National instruments).

Light measurements were performed using a calibrated spectroradiometer (Bentham instruments, Reading, UK). LED intensity was initially calibrated (using the principles described above) to recreate for *Opn1mw* ^R^ individuals the effective rod, cone and melanopsin excitation experienced by a wild-type (green cone) mouse visual system under typical natural daylight (average values from our environmental data above at a solar angle 3° above the horizon; S1A Fig). We also carefully calibrated differential modulations in the intensity of each LED to produce stimuli that independently varied in apparent brightness for one or both cone opsin classes (either in unison or antiphase) with no apparent change in rod or melanopsin excitation (S1B Fig). In each case, brightness for the stimulated opsin was varied by ±70%, to produce an overall 4.7-fold increase in intensity of between “bright” and “dim” phases of the stimulus. Transitions between the two stimulus phases occurred smoothly over 50ms (half sinusoid profile). We also applied stimuli that selectively modulated melanopsin excitation (±92%), without changing effective cone excitation. These later also, in principle, modulated apparent brightness for rod photoreceptors (±84%), however we think a rod contribution to the resulting responses unlikely owing to the high background light levels (14.9 rod effective photons/cm^2^/s) and our previous work suggesting that rods have little influence on acute electrophysiological light responses in the SCN [8]. Indeed, similar stimuli evoke very little response in the lateral geniculate nuclei of melanopsin knockout animals [22].

In a subset of experiments (7/15) we also applied a second set of stimuli designed to recreate various stages of twilight, using our calculations of the effective photon fluxes experienced by mouse opsins at solar angles between -7 and 3° relative to the horizon. These were applied as light steps (30 s) from darkness in random sequence with an interstimulus interval of 2 min. To confirm whether elements of the resulting responses were dependent on spectral composition, these stimuli were interspersed with two additional stimulus sets which were identical except that irradiance for the UVS opsin was fixed at a constant ratio relative to LWS (mimicking either day or night spectral composition).

For behavioural experiments, we used similar principles to generate photoperiods that smoothly recreated our measured changes in twilight illumination, with (“natural”) or without the associated change in spectral composition (irradiance-only: spectra fixed to mimic “night”). Stimuli were generated by an array of three violet (400 nm) and three amber (590 nm) high-power LEDs (LED Engin Inc., San Jose CA, US) placed behind a polypropylene diffusing screen covering the top of the cage. The combination of multiple LEDs allowed a larger range of brightness (from dark up to approximately 25 W/m2 for the violet and 10 W/m2 for the amber). Intensity of each LED was independently controlled by a voltage controlled driver (Thorlabs Inc., Newton NJ, US). The light intensity modulation signals were provided by a PC running Labview through a voltage output module (National Instruments), and followed a temporal profile that recreated the sun’s progression during a northern latitude summer (calculations based on Stockholm, Sweden; Lat: 59, Long: 18, Elevation 76 m, 20 June 2013; total twilight duration = 2.3 h).

### Twilight Entrainment Study

To determine the impact of twilight spectral changes on mouse entrainment, female *Opn1mw* ^*R*^ *and Cnga3* ^*-/-*^ mice (housed under an 18:6 light–dark [LD] cycle) were first implanted with iButton temperature loggers (Maxim, DS1922L-F5#). To reduce weight and size, these were dehoused and encapsulated in a 20% Poly(ethylene-co-vinyl acetate) and 80% paraffin mixture as described by Lovegrove [47]. For implantation, mice were anaesthetised with isoflurane (1%–5% in O_2_) and the temperature logger implanted into the peritoneal cavity. Following surgery, animals were given a 0.03 mg/kg subcutaneous dose of buprenorphine and allowed to recover for at least 9 d in 18:6 LD before the start of the experiment. The timing of lights off under this cycle was designated as *Zeitgeber* time (ZT) 12 and the timing of experimental photoperiods were set to align their midnight (ZT15) with this square wave LD cycle.

Following recovery, group housed mice (five per cage) were transferred to the natural twilight photoperiod. The cage environment contained an opaque plastic hide, allowing the animals to choose their own light sampling regime. After 14 d, mice were then returned to 18:6 LD for a further 14 d and finally transferred to the “irradiance-only” twilight photoperiod.

At the end of the experiment, mice were culled by cervical dislocation and temperature loggers recovered. Temperature data (recorded in 30 min time bins) was processed by upsampling to 5 min resolution (cubic spline interpolation), Gaussian smoothing (SD = 45 min), and normalisation as a fraction of daily temperature range. Phase of entrainment was estimated as the timing of peak body temperature from that individual’s daily average profile (calculated from the last 9 d in each photoperiod).

### Ex Vivo SCN Recordings

*Opn1mw* ^*R*^ *and Cnga3* ^*-/-*^ mice were housed under twilight stimuli of either “natural” or “night” composition (as described above) for at least 14 d prior to experiments. Mice were removed from the home cage 30–60 min after the end of the dawn transition (~ZT19) and culled by cervical dislocation followed by decapitation. The brain was then rapidly removed, mounted onto a metal stage and cut using a 7000 smz vibrating microtome (Campden Instrument, UK) in ice-cold (~4°C) sucrose-based slicing solution composed of (in mM): sucrose (189); D-glucose (10); NaHCO_3_ (26); KCl (3); MgSO_4_ (5); CaCl_2_ (0.1); NaH_2_PO_4_ (1.25); oxygenated with 95% O_2_/5% CO_2_ mixture. Coronal brain slices containing the SCN (350 μm) were then immediately transferred into a petri dish containing oxygenated artificial cerebrospinal fluid (aCSF) composed of (in mM): NaCl (124); KCl (3); NaHCO_3_ (24); NaH_2_PO_4_ (1.25); MgSO_4_ (1); glucose (10); CaCl_2_ (2); slices were then left to rest at room temperature (22 ± 1°C).

Approximately 30 min after slice preparation, slices were placed, recording side down, onto 6x10 perforated multielectrode arrays (pMEAs; Multichannel Systems, MCS, Germany). Slices were visualised under the microscope and photos were taken with a GXCAM-1.3 camera (GX Optical, UK) in order to confirm appropriate slice placement over pMEA electrode sites. Slices were held in place by both the suction via the MEA perforations and a harp slice grid (ALA Scientific Instruments Inc., US). The pMEA recording chamber was continuously perfused with pre-warmed oxygenated aCSF (34 ± 1°C) to both slice surfaces at a rate of 2.5–3 ml/min. Neural signals were acquired as time-stamped action potential waveforms using a USB-ME64 system and a MEA1060UP-BC amplifier (MCS, Germany). Signals were sampled at 12.5 kHz, High pass filtered at 200 Hz (second order Butterworth) with a threshold of usually at -16.5 μV. Recordings were maintained for a total duration of 30 h.

At the end of each recording, slices were treated with bath applications of 20μM NMDA to confirm maintained cell responsiveness, followed by 1 μM TTX to confirm acquired signals exclusively reflected Na^+^-dependent action potentials. All drugs were purchased from Tocris (UK), kept as stock solutions at -20°C (dissolved in dH_2_O), and were diluted to their respective final concentrations directly in pre-warmed, oxygenated aCSF; all drugs were bath applied for 5 min.

Multiunit action potential firing rates detected at electrodes located within the SCN region were then selected for further analysis. Data were subsequently binned (60 s) and smoothed via boxcar averaging (width: 2 h) to determine the timing of peak activity. Channels where peak firing did not decay by >50% within ±12 h, or where peak firing was less than 0.2 spikes/s were excluded from this analysis, such that on average 22 ± 2 SCN electrodes were analysed for each experiment. Based on peak firing rates observed (mean ± SEM: 6.9 ± 0.5 spikes/s) we estimate these typically represent recordings from less than four neurons.

To assess for significant differences in the timing of population activity under our different experimental conditions, we drew 1,000 samples of 100 randomly selected neurons from each condition (*Opn1mw* ^*R*^ “natural”, *Opn1mw* ^*R*^ “night”, *Cnga3* ^*-/-*^ “natural”). By calculating the circular mean phase for each sample, we thus obtained estimates of the probability that the observed population means differed by chance.

## Supporting Information

S1 Data Excel spreadsheet containing, in separate sheets, the numerical data and statistical analysis for Fig 1A–1D and underlying raw values used to generate those averages (total optical power, colour index, MWS and UVS opsin flux versus solar angle for each observation). (XLSX)

S2 Data Excel spreadsheet containing, in separate sheets, the numerical data and statistical analysis for Figs 2A–2C and S2C and S2D and underlying raw values used to generate averages (peristimulus time histograms for all blue ON, yellow ON, and achromatic cells responses to each stimulus condition). (XLSX)

S3 Data Excel spreadsheet containing, in separate sheets, the numerical data and statistical analysis for Fig 3A and 3B and underlying raw data values (peristimulus time histograms for all blue ON, yellow ON, and achromatic cells responses to each stimulus condition). (XLSX)

S4 Data Excel spreadsheet containing, in separate sheets, the numerical data and statistical analysis for Figs 4A–4E and S3A–S3C and underlying raw values used to generate averages (peristimulus time histograms for all blue ON and achromatic cells responses to each stimulus condition and “unclassified” cell responses to natural twilight). (XLSX)

S5 Data Excel spreadsheet containing the numerical data and statistical analysis for Fig 5A and 5B and underlying raw values used to generate averages (daily body temperature profiles for *Opn1mw* ^*R*^ and *cnga3* ^*-/-*^ mice under both photoperiods). (XLSX)

S6 Data Excel spreadsheet containing, in separate sheets, the numerical data and statistical analysis for Figs 6A and 6B and S5A and sampled distributions used to assess significant differences in phase. (XLSX)

S1 Fig Selective modulation of colour and brightness in red cone knock-in mice. (**A**) Top panel shows normalised spectral profile of daytime ambient illumination (blue line; +3°) alongside sensitivity profiles for native mouse opsins. Numbers above traces indicate the calculated photon flux for each photoreceptor class (Log photons/cm^2^/s). Bottom panel shows spectral profile of three-primary LED system used to recreate natural daylight from red cone knock-in mice (*Opn1mw* ^R^). Note the shift in cone sensitivity from MWS (top panel) to LWS (bottom panel): the photon flux experienced by wild-type mouse MWS cones (15 log photons/cm2/s) is translated here into an equivalent photon flux for the LWS cone knock-in. (**B**) Illustration of spectral modulations used to selectively evoke changes in relative (“colour”; top) or absolute (“brightness”; bottom) activation of UV and long-wavelength sensitive (UVS/LWS) cone opsins. Numbers on traces reflect Log photon flux at blue versus yellow (top panel) or dim versus bright (bottom panel) stimulus phases. These equate to 4.7-fold changes (70% Michelson contrast) in cone opsin activation with essentially no effective change in melanopsin excitation (<1% Michelson). Effective changes in rod excitation are omitted for clarity but are also very small (6.5% and 4.5% Michelson for “brightness” and “colour” respectively), however, owing to the overall intensity of the stimuli, it is highly unlikely that rods could contribute to any response even with much larger contrasts.
(TIF)

S2 Fig Effect of background spectra on SCN spectral sensitivity. (**A**) Spectral modulations used to selectively evoke changes in LWS (top) or UVS opsin excitation (bottom) under “natural” daylight and a “white” background where basal activation of UVS and LWS opsin were equivalent. In each case, effective change for the modulated opsin is 70% Michelson contrast (non-modulated (“silent”) opsins <1% Michelson). Note that, since most mouse cones co-express both opsins (and must sum over all photons detected), the net change in cone photon flux presented by these stimuli is influenced both by the relative background photon flux at each of the two opsin classes and by the degree of co-expression in each cone. (**B**) Model of the effective change in irradiance presented by cone opsin-isolating stimuli, as a function of relative opsin co-expression, under “natural” and “white” backgrounds. (**C**: top) Normalised mean (±SEM) responses to UVS/LWS contrast under natural and white background spectra for all blue-ON and yellow-ON cells (*n* = 13 and 4 respectively). Shading indicates “dim” to “bright” transition, *x*-axis scale bars indicate temporal profile of UVS/LWS opsin excitation. Note, LWS and UVS opsin specific responses are modulated by changes in background spectra consistent with the involvement of cone that co-express both opsins. (**C**: bottom) Example responses of two colour-sensitive cells whose opponency was highly dependent on background spectra (one other blue-ON cell exhibited similar behaviour, not shown). *Y*-axis scale bar indicates firing frequency in spikes/s. (**D**) Example achromatic cell response (top) and normalised population mean (±SEM; bottom) for UVS and LWS contrast under the two backgrounds. The data used to make this figure can be found in S2 Data.
(TIF)

S3 Fig Responses of SCN neurons to simulated twilight. (**A**: top) Mean (±SEM) normalised responses (5s bins) of blue-ON (left; *n* = 9) and achromatic units (right; *n* = 8) to stimuli with “natural” or “day” spectra at intensities corresponding to dawn (solar angle 0.5° above horizon; spectra 4 from Fig 4A). Data analysed by one-way RM-ANOVA with Sidak post-hoc test; ** *p*<0.01, * *p*<0.05. Bottom plots show mean response time course. Note the significantly larger responses of blue-ON cells to “natural” versus “day” stimuli despite only very small differences in the effective UVS opsin excitation produced by these two stimuli (~5%, 0.02 log units). (**B**) Steady state firing rates in darkness and under “day-time” illumination (equivalent to solar angle of 3° above horizon; last 10 s of 30 s light step) for various light-responsive SCN cell classes. Data analysed by paired *t* test between light and dark, *** *p*<0.001. (**C**) Mean (±SEM) normalised responses of SCN cells that could not be classified as brightness/colour sensitive (*n* = 25) to 30 s light steps recreating various stages of twilight. These were qualitatively similar to those of brightness sensitive cells (Fig 4D and 4E). The data used to make this figure can be found in S3 Data.
(TIF)

S4 Fig Assessing influences of twilight spectra on circadian entrainment. (**A**) Schematic of housing conditions for experiments assessing the influence of twilight spectra. Mice (with temperature sensors implanted i.p.) were group housed (5/cage) and uniform illumination applied across the entire top surface of the cage via an artificial sky (comprising multiple amber and violet LEDs controlled by pulse width modulation). Mice could freely move between the open cage and an opaque plastic hide, allowing individuals to choose their own light sampling regimen. (**B**) Artificial sky was calibrated to replicate, for *Opn1mw* ^R^ individuals, a wild-type mouse’s experience of twilight (left: “natural”). A second, analytical twilight stimulus was calibrated to match the natural change in irradiance but with relative activation of cone opsins fixed to match the night spectra (right: “night”). (**C**) To maximise our ability to distinguish differences in phase of entrainment under the above conditions, twilight stimuli were set to a cycle that simulated the sun’s progression during a northern latitude summer (calculations based on Stockholm, Sweden; Lat.: 59, Long.: 18, Elevation 76 m, 20 June 2013) for a total ““twilight”“duration of 2.3 h.
(TIF)

S5 Fig Photoperiodic encoding in the suprachiasmatic nuclei of coneless mice. Phasing of SCN firing rhythms from ex vivo multielectrode array recordings of *Cnga3* ^*-/-*^ mice housed under a simulated natural twilight photoperiod. Left panel shows Rayleigh vector plots for peak firing activity (*n* = 103 SCN electrodes from five slices). Grey shaded area corresponds to timing of night/twilight transitions, red dotted lines indicate central 50% of the data distribution, arrow indicates mean vector direction. Right panels show representative multiunit traces. Consistent with body temperature data (Fig 5), SCN activity peaks earlier in *Cnga3* ^*-/-*^ mice relative to Opn1mwR animals housed under “natural” twilight (Fig 6A; *p*<0.001 based on bootstrap percentiles). The data used to make this figure can be found in S6 Data.
(TIF)

S6 Fig Anatomical locations of visually responsive cell types in the suprachiasmatic nuclei. Projected anatomical locations of the visually responsive SCN cells reported in this study, split according to response type. The Unclassified population corresponds to cells that responded to light steps from darkness but not to cone-isolating stimuli.
(TIF)

## Abbreviations

ipRGCs: intrinsically photosensitive retinal ganglion cells
LD: light–dark
LWS: long-wavelength sensitive
MWS: medium wavelength sensitive
SCN: suprachiasmatic nuclei
UVS: ultraviolet sensitive
