# Supplementary figures and images for: Colour As a Signal for Entraining the Mammalian Circadian Clock
Source: PLoS Biol. 2015 Apr 17;13(4):e1002127. doi: 10.1371/journal.pbio.1002127 (PMC4401556; doi:10.1371/journal.pbio.1002127)

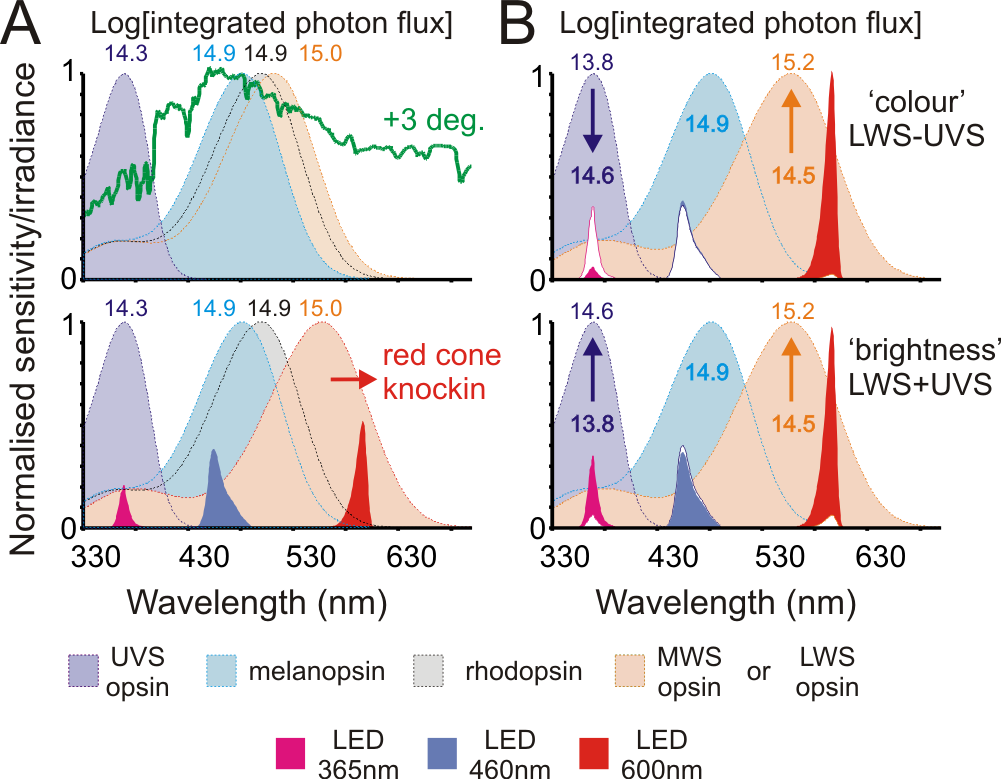

Supplement: S1 Fig — (A) Top panel shows normalised spectral profile of daytime ambient illumination (blue line; +3°) alongside sensitivity profiles for native mouse opsins. Numbers above traces indicate the calculated photon flux for each photoreceptor class (Log photons/cm2/s). Bottom panel shows spectral profile of three-primary LED system used to recreate natural daylight from red cone knock-in mice (Opn1mw R). Note the shift in cone sensitivity from MWS (top panel) to LWS (bottom panel): the photon flux experienced by wild-type mouse MWS cones (15 log photons/cm2/s) is translated here into an equivalent photon flux for the LWS cone knock-in. (B) Illustration of spectral modulations used to selectively evoke changes in relative (“colour”; top) or absolute (“brightness”; bottom) activation of UV and long-wavelength sensitive (UVS/LWS) cone opsins. Numbers on traces reflect Log photon flux at blue versus yellow (top panel) or dim versus bright (bottom panel) stimulus phases. These equate to 4.7-fold changes (70% Michelson contrast) in cone opsin activation with essentially no effective change in melanopsin excitation (<1% Michelson). Effective changes in rod excitation are omitted for clarity but are also very small (6.5% and 4.5% Michelson for “brightness” and “colour” respectively), however, owing to the overall intensity of the stimuli, it is highly unlikely that rods could contribute to any response even with much larger contrasts. (TIF) [file pbio.1002127.s007.tif]

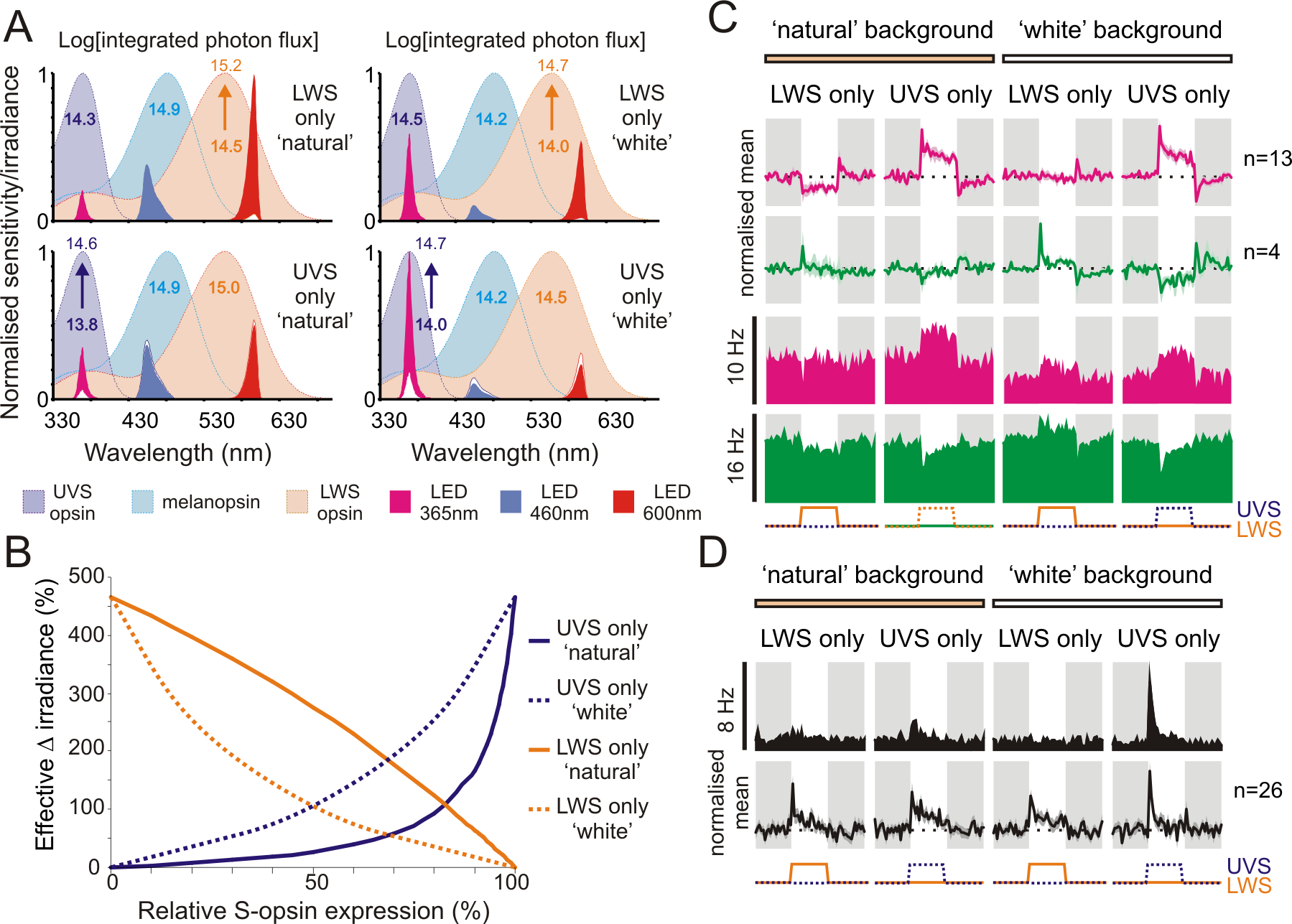

Supplement: S2 Fig — (A) Spectral modulations used to selectively evoke changes in LWS (top) or UVS opsin excitation (bottom) under “natural” daylight and a “white” background where basal activation of UVS and LWS opsin were equivalent. In each case, effective change for the modulated opsin is 70% Michelson contrast (non-modulated (“silent”) opsins <1% Michelson). Note that, since most mouse cones co-express both opsins (and must sum over all photons detected), the net change in cone photon flux presented by these stimuli is influenced both by the relative background photon flux at each of the two opsin classes and by the degree of co-expression in each cone. (B) Model of the effective change in irradiance presented by cone opsin-isolating stimuli, as a function of relative opsin co-expression, under “natural” and “white” backgrounds. (C: top) Normalised mean (±SEM) responses to UVS/LWS contrast under natural and white background spectra for all blue-ON and yellow-ON cells (n = 13 and 4 respectively). Shading indicates “dim” to “bright” transition, x-axis scale bars indicate temporal profile of UVS/LWS opsin excitation. Note, LWS and UVS opsin specific responses are modulated by changes in background spectra consistent with the involvement of cone that co-express both opsins. (C: bottom) Example responses of two colour-sensitive cells whose opponency was highly dependent on background spectra (one other blue-ON cell exhibited similar behaviour, not shown). Y-axis scale bar indicates firing frequency in spikes/s. (D) Example achromatic cell response (top) and normalised population mean (±SEM; bottom) for UVS and LWS contrast under the two backgrounds. The data used to make this figure can be found in S2 Data. (TIF) [file pbio.1002127.s008.TIF]

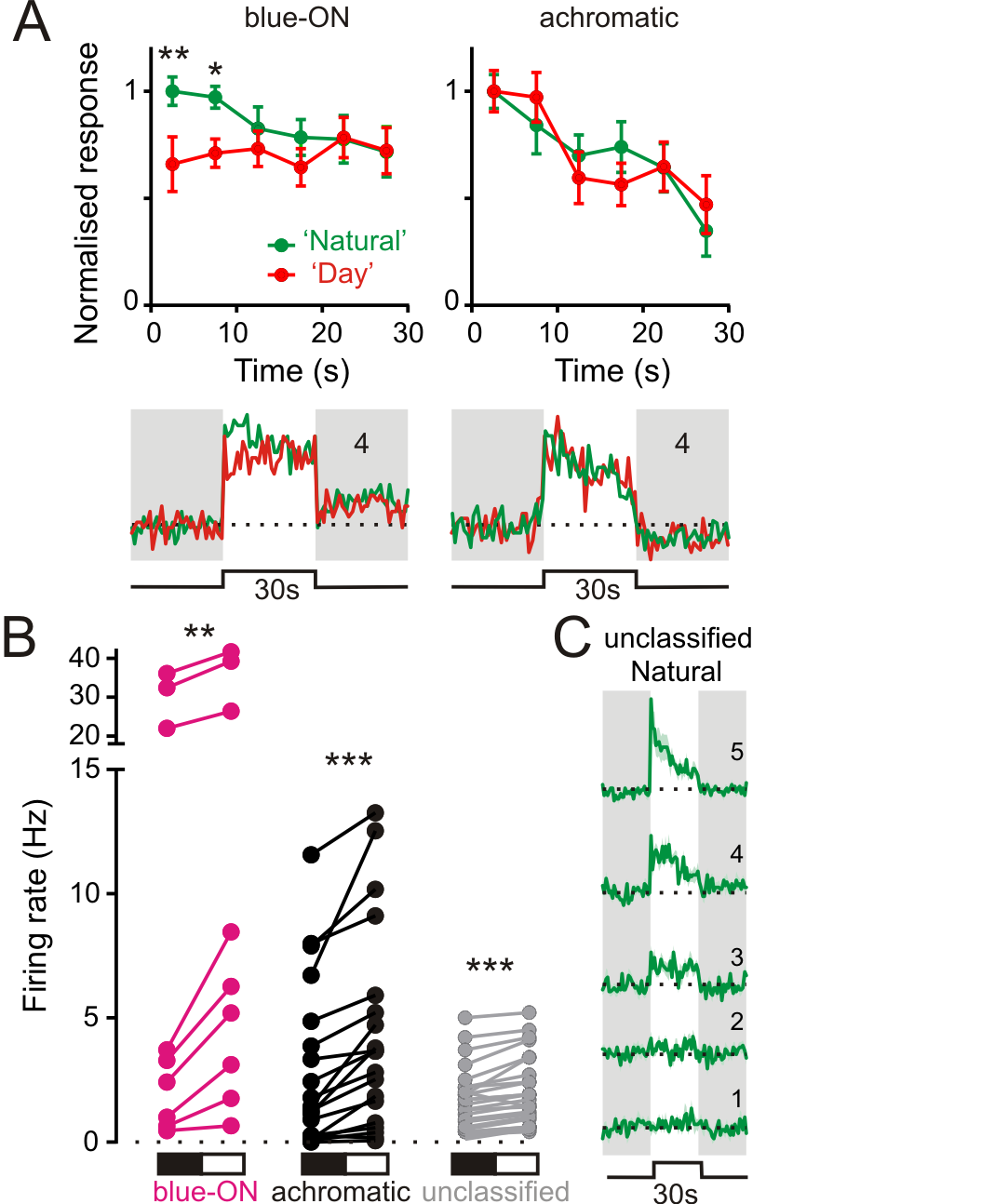

Supplement: S3 Fig — (A: top) Mean (±SEM) normalised responses (5s bins) of blue-ON (left; n = 9) and achromatic units (right; n = 8) to stimuli with “natural” or “day” spectra at intensities corresponding to dawn (solar angle 0.5° above horizon; spectra 4 from Fig 4A). Data analysed by one-way RM-ANOVA with Sidak post-hoc test; ** p<0.01, * p<0.05. Bottom plots show mean response time course. Note the significantly larger responses of blue-ON cells to “natural” versus “day” stimuli despite only very small differences in the effective UVS opsin excitation produced by these two stimuli (~5%, 0.02 log units). (B) Steady state firing rates in darkness and under “day-time” illumination (equivalent to solar angle of 3° above horizon; last 10 s of 30 s light step) for various light-responsive SCN cell classes. Data analysed by paired t test between light and dark, *** p<0.001. (C) Mean (±SEM) normalised responses of SCN cells that could not be classified as brightness/colour sensitive (n = 25) to 30 s light steps recreating various stages of twilight. These were qualitatively similar to those of brightness sensitive cells (Fig 4D and 4E). The data used to make this figure can be found in S3 Data. (TIF) [file pbio.1002127.s009.TIF]

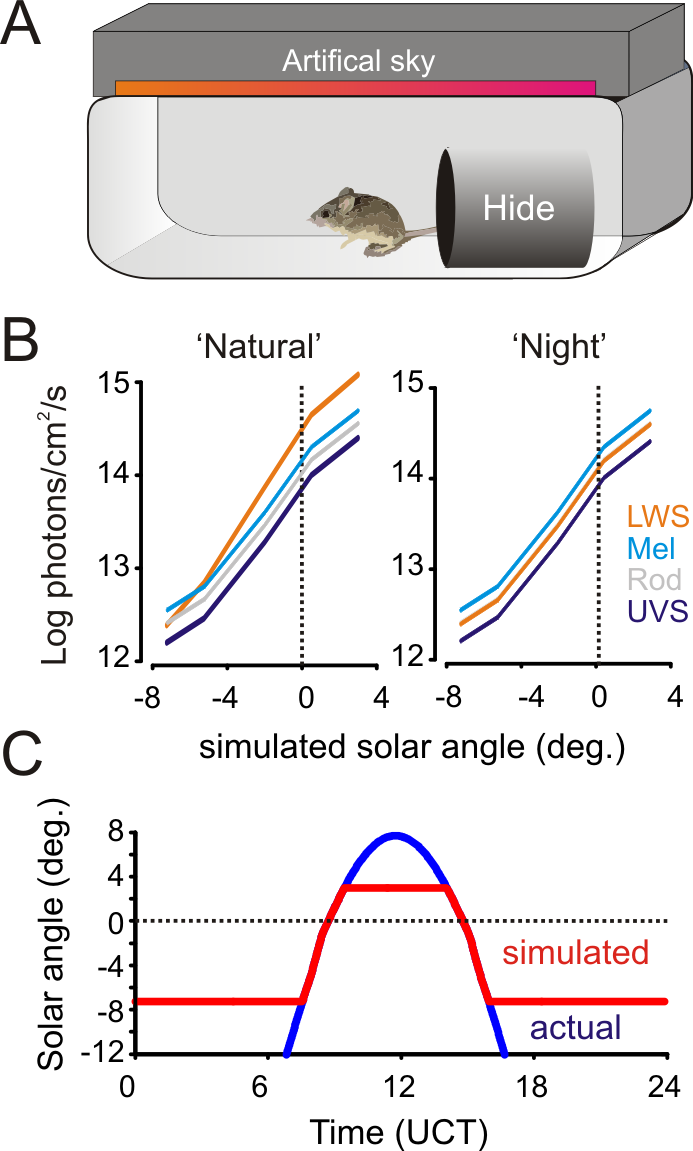

Supplement: S4 Fig — (A) Schematic of housing conditions for experiments assessing the influence of twilight spectra. Mice (with temperature sensors implanted i.p.) were group housed (5/cage) and uniform illumination applied across the entire top surface of the cage via an artificial sky (comprising multiple amber and violet LEDs controlled by pulse width modulation). Mice could freely move between the open cage and an opaque plastic hide, allowing individuals to choose their own light sampling regimen. (B) Artificial sky was calibrated to replicate, for Opn1mw R individuals, a wild-type mouse’s experience of twilight (left: “natural”). A second, analytical twilight stimulus was calibrated to match the natural change in irradiance but with relative activation of cone opsins fixed to match the night spectra (right: “night”). (C) To maximise our ability to distinguish differences in phase of entrainment under the above conditions, twilight stimuli were set to a cycle that simulated the sun’s progression during a northern latitude summer (calculations based on Stockholm, Sweden; Lat.: 59, Long.: 18, Elevation 76 m, 20 June 2013) for a total ““twilight”“duration of 2.3 h. (TIF) [file pbio.1002127.s010.TIF]

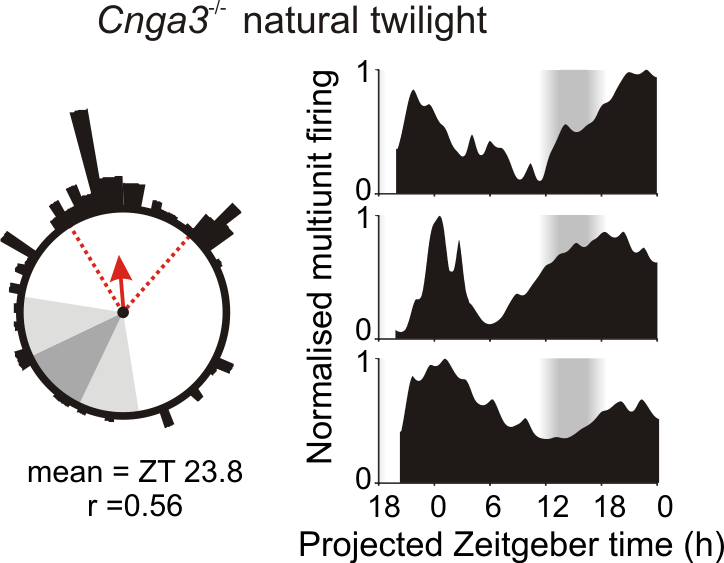

Supplement: S5 Fig — Phasing of SCN firing rhythms from ex vivo multielectrode array recordings of Cnga3 -/- mice housed under a simulated natural twilight photoperiod. Left panel shows Rayleigh vector plots for peak firing activity (n = 103 SCN electrodes from five slices). Grey shaded area corresponds to timing of night/twilight transitions, red dotted lines indicate central 50% of the data distribution, arrow indicates mean vector direction. Right panels show representative multiunit traces. Consistent with body temperature data (Fig 5), SCN activity peaks earlier in Cnga3 -/- mice relative to Opn1mwR animals housed under “natural” twilight (Fig 6A; p<0.001 based on bootstrap percentiles). The data used to make this figure can be found in S6 Data. (TIF) [file pbio.1002127.s011.tif]

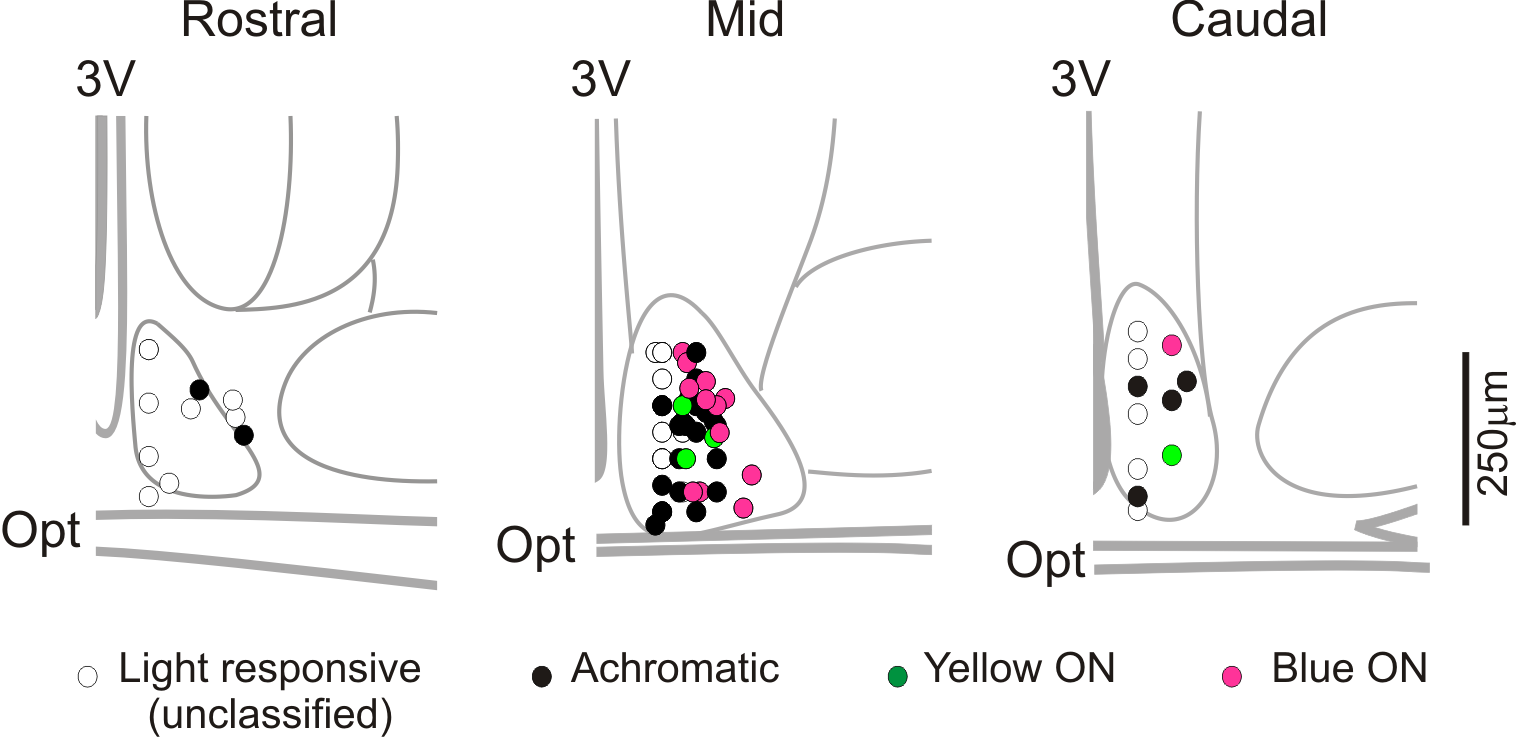

Supplement: S6 Fig — Projected anatomical locations of the visually responsive SCN cells reported in this study, split according to response type. The Unclassified population corresponds to cells that responded to light steps from darkness but not to cone-isolating stimuli. (TIF) [file pbio.1002127.s012.tif]
